# Activation of Nrf2 by MIND4-17 protects osteoblasts from hydrogen peroxide-induced oxidative stress

**DOI:** 10.18632/oncotarget.22360

**Published:** 2017-11-10

**Authors:** Shiguang Guo, Hao-Dong Fei, Feng Ji, Feng-Li Chen, Yue Xie, Shou-Guo Wang

**Affiliations:** ^1^ Department of Intensive Care Unit, Huai'an First People's Hospital, Nanjing Medical University, Huai'an, China; ^2^ Department of Orthopedics, Huai'an First People's Hospital, Nanjing Medical University, Huai'an, China; ^3^ Clinical Laboratory, Huai'an First People's Hospital, Nanjing Medical University, Huai'an, China

**Keywords:** osteoblasts, oxidative stress, Nrf2, MIND4-17, Keap

## Abstract

MIND4-17 is a recently developed NF-E2-related factor 2 (Nrf2) activator, which uniquely causes Nrf2 disassociation from Keap1. Here, we showed that pretreatment with MIND4-17 significantly inhibited hydrogen peroxide (H_2_O_2_)-induced viability reduction of primary osteoblasts and OB-6 osteoblastic cells. Meanwhile, MIND4-17 inhibited both apoptotic and non-apoptotic osteoblast cell death by H_2_O_2_. MIND4-17 treatment induced Keap1-Nrf2 disassociation, causing Nrf2 stabilization, accumulation and nuclear translocation in osteoblasts, leading to transcription of several Nrf2-dependent genes, including *heme oxygenase 1 (HO-1), NAD(P)H quinone oxidoreductase 1 (NQO1), γ-glutamylcysteine synthetase modifier subunit (GCLM)* and *catalytic subunit (GCLC)*. Additionally, MIND4-17 largely attenuated H_2_O_2_-reactive oxygen species (ROS) production, lipid peroxidation and DNA damages. Nrf2 knockdown by targeted short hairpin RNA (shRNA) exacerbated H_2_O_2_-induced cytotoxicity in OB-6 osteoblastic cells, and nullified MIND4-17-mediated cytoprotection against H_2_O_2_. Meanwhile, Keap1 shRNA took over MIND4-17′s actions and protected OB-6 cells from H_2_O_2_. Together, MIND4-17 activates Nrf2 signaling and protects osteoblasts from H_2_O_2_.

## INTRODUCTION

Osteonecrosis is a common cause of bone fracture [1, 2], which is often accompanied with increased oxidative stress in the bone [3, 4]. Osteoblasts are bone's mesenchymal progenitor cells-derived cells, which are vital for the bone formation and remodeling [1, 2]. Increased reactive oxygen species (ROS) production and subsequent oxidative stress shall exert toxicities to the osteoblasts, causing lipid peroxidation, DNA damages and cell apoptotic/non-apoptotic death [1, 2, 5–7]. Hydrogen peroxide (H_2_O_2_) is often added to cultured osteoblasts to mimic oxidative injuries [8–11].

NF-E2-related factor 2 (Nrf2) is one of the most studied cellular defense mechanisms against oxidative stress [12–14]. Keap1 (Kelch-like erythroid cell-derived protein with CNC homology [ECH]-associated protein 1) is the key regulator and repressor protein of Nrf2 [12, 13]. Keap1 association with Nrf2 causes Nrf2 ubiquitination and degradation [12, 13]. Reversely, Keap1 silence, mutation or inhibition shall induce Nrf2 stabilization and accumulation [15, 16]. Afterwards, Nrf2 will translocate to cell nuclei to promote transcription of multiple anti-oxidant genes via binding to antioxidant response element (ARE) [12, 13]. Multiple Nrf2-dependnet anti-oxidant genes have been demonstrated, including *heme oxygenase 1 (HO-1), NAD(P)H quinone oxidoreductase 1 (NQO1)*, *γ-glutamylcysteine synthetase modifier subunit (GCLM)* and *catalytic subunit (GCLC)*, along with others [12, 13].

Previous studies have shown that Nrf2 signaling activation could efficiently protect osteoblasts from oxidative stresses [17, 18]. Recent research has developed a novel thiazole-containing compound targeting Nrf2, which was named as MIND4-17 [19, 20]. This compound uniquely modifies Keap1′s stress-sensor cysteine (C151), leading to Keap1 protein conformation change [19, 20]. This would lead to Keap1-Nrf2 disassociation, causing Nrf2 stabilization, accumulation and nuclear translocation [19, 20]. Thus, MIND4-17 uniquely and efficiently induces Nrf2 activation [19, 20]. In the present study, we showed that activation of Nrf2 by MIND4-17 protected osteoblasts from H_2_O_2_.

## RESULTS

### MIND4-17 protects human osteoblasts from hydrogen peroxide

**Figure 1:**
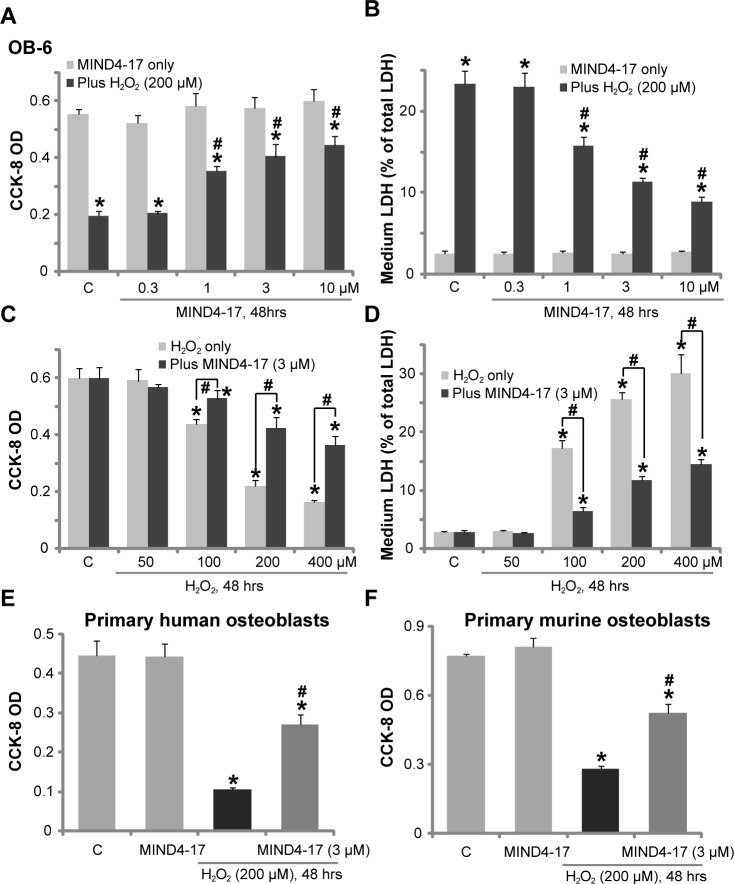
MIND4-17 protects human osteoblasts from hydrogen peroxide OB-6 human osteoblastic cells (**A**-**D**), the primary human osteoblasts (**E**) or the primary murine osteoblasts (**F**) were pretreated for 1 hour with applied concentration of MIND4-17, following by the hydrogen peroxide (“H_2_O_2_”) treatment, cells were further cultured for additional 48 hours in the conditional medium; Cell viability (CCK-8 assay, A, C, E and F) and cell death (LDH release assay, B and D) were tested. Data were presented as mean (n=5) ± standard deviation (SD). “C” stands for medium treatment control (Same for all figures). ^*^p<0.05 *vs.* “C” cells. ^#^*p*<0.05 *vs.* H_2_O_2_ only group. Experiments in this figure were repeated for four times, and similar results were obtained.

The *in vitro* studies added H_2_O_2_ to cultured osteoblasts to mimic oxidative damages [17, 21, 22]. Here, we found that treatment the human osteoblastic OB-6 cells [23] with H_2_O_2_ (200 μM) for 48 hours induced profound viability [Cell Counting Kit-8 (CCK-8) optic density (OD)] reduction (Figure 1A) and cell death [Lactate Dehydrogenase (LDH) release, Figure 1B]. Remarkably, pretreatment (for 1 hour) with MIND4-17, the Nrf2-inducing small molecule compound [19, 20], attenuated H_2_O_2_-induced OB-6 cell death (Figure 1A and 1B). Notably, MIND4-17-induced cytoprotection against H_2_O_2_ was dose-dependent, and its effect was only significant at 1-10 μM (Figure 1A and 1B). MIND4-17 treatment alone at the tested concentrations (0.3-10 μM) failed to change OB-6 cell survival and death (Figure 1A and 1B).

OB-6 cells were also treated with H_2_O_2_ at other concentrations, from 50-400 μM. With the increase of H_2_O_2_'s concentration, more cell death was noticed (Figure 1C and 1D). Pretreatment with 3 μM of MIND4-17 was again cytoprotective, and inhibited cytotoxicity by H_2_O_2_ at all tested concentrations (Figure 1C and 1D). We also tested the effect of this novel Nrf2 inducer in other osteoblasts. As displayed, in both primary human osteoblasts (Figure 1E) and primary murine osteoblasts (Figure 1F), pretreatment with MIND4-17 (3 μM, 1 hour) potently inhibited H_2_O_2_ (200 μM)-induced cell viability (CCK-8 OD) reduction. Collectively, these results demonstrate that MIND4-17 protects human osteoblasts from H_2_O_2_.

### MIND4-17 inhibits H_2_O_2_-induced apoptotic and non-apoptotic cell death of osteoblasts

**Figure 2:**
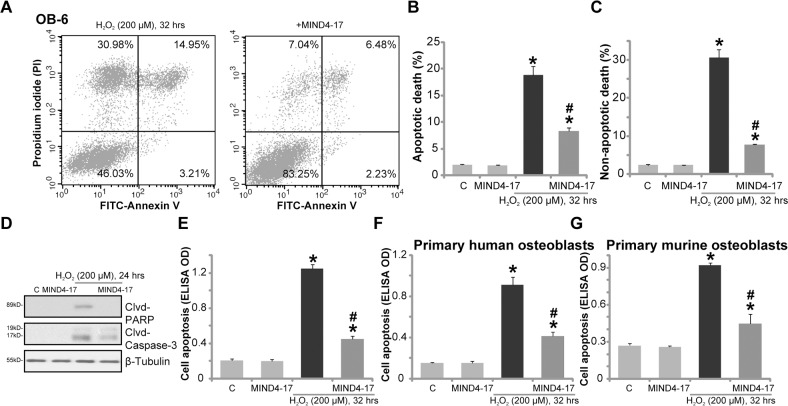
MIND4-17 inhibits H_2_O_2_-induced apoptotic and non-apoptotic cell death of osteoblasts OB-6 human osteoblastic cells (**A**-**E**), the primary human osteoblasts (**F**) or the primary murine osteoblasts (**G**) were pretreated for 1 hour with MIND4-17 (3 μM), following by hydrogen peroxide (“H_2_O_2_”, 200μM) treatment, cells were then cultured for the indicated time period and were subjected to assays mentioned in the text to examine apoptotic and non-apoptotic cell death. Data were presented as mean (n=5) ± standard deviation (SD). ^*^p<0.05 *vs.* “C” cells. ^#^*p*<0.05 *vs.* H_2_O_2_ only group. Experiments in this figure were repeated for three times, and similar results were obtained.

H_2_O_2_ is shown to induce both apoptotic and non-apoptotic cell death [24–26], the latter is mediated through mitochondria, which is also known as “programmed necrosis” [24–26]. In line with these findings, our results from the Fluorescence Activated Cell Sorting (FACS) analysis confirmed that H_2_O_2_ indeed simultaneously induced apoptotic (Annexin V^+/+^) and non-apoptotic (Annexin V^−/−^ and PI^+/+^) OB-6 cell death (Figure 2A-2C). Significantly, H_2_O_2_-induced apoptosis and non-apoptotic cell death were both largely inhibited by pretreatment of MIND4-17 (3 μM, 1 hour) (Figure 2A-2C). OB-6 cell apoptosis by H_2_O_2_ treatment was further confirmed by cleavages of caspase-3 and PARP [poly (ADP-ribosyl) transferase] (Figure 2D), as well as increase of histone DNA apoptosis ELISA OD (Figure 2E). MIND4-17 pretreatment again largely attenuated above pro-apoptosis activity by H_2_O_2_ (Figure 2D and 2E). Treatment with MIND4-17 alone in OB-6 cells had no significant effect on cell apoptotic /non-apoptotic death (Figure 2A-2E). In the primary human osteoblasts (Figure 2F) and primary murine osteoblasts (Figure 2G), H_2_O_2_ (200 μM)-induced cell apoptosis, reflected by histone DNA apoptosis ELISA OD increase, was also largely inhibited by MIND4-17 (3 μM). Together, MIND4-17 inhibits H_2_O_2_-induced apoptotic and non-apoptotic cell death of osteoblasts.

### MIND4-17 activates Nrf2 signaling in osteoblasts

**Figure 3:**
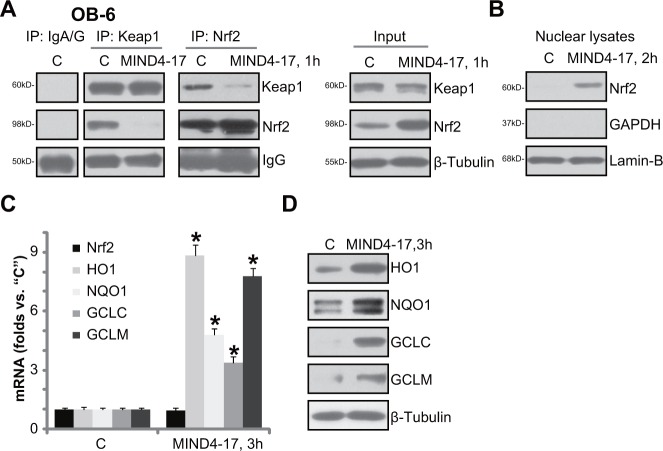
MIND4-17 activates Nrf2 signaling in osteoblasts OB-6 human osteoblastic cells were treated with MIND4-17 (3 μM) for applied time; The association between Nrf2 and Keap1 was examined by the co-immunoprecipitation (“Co-IP”) assay (A, left panel); Expressions of the indicated proteins in total cell lysates (**A**, right panel, and **D**) as well as in the nuclear lysates (**B**, Lamin-B was a marker protein) were presented; *mRNA* expressions of listed genes were tested by quantitative real-time PCR (“qRT-PCR”) assay (**C**). Data were presented as mean (n=5) ± standard deviation (SD). ^*^p<0.05 *vs.* “C” cells. Experiments in this figure were repeated for three times, and similar results were obtained.

The potential effect of MIND4-17 on Nrf2 signaling was also tested. First, co-immunoprecipitation (“Co-IP”) assay was performed to test the association between Nrf2 and its inhibitor protein Keap1 [27–29]. As shown in Figure 3A (IP results) MIND4-17 (3 μM, 1 hour) treatment almost completely blocked Nrf2-Keap1 association in OB-6 cells. Input assay of in Figure 3A (“Input”) confirmed MIND4-17 induced Nrf2 protein accumulation, yet Keap1 protein level was unchanged. Subsequently, the MIND4-17-stabilized Nrf2 translocated to cell nuclei (Figure 3B, analyzing nuclear fraction proteins). Afterwards, *mRNA* expressions of Nrf2-ARE-dependent genes, including *HO1, NQO1, GCLC* and *GCLM* were all increased following MIND4-17 treatment (Figure 3C). Meanwhile, protein expressions of the Nrf2-dependent anti-oxidant genes (HO1, NQO1, GCLC and GCLM) were also boosted (Figure 3D). It should be noted that *Nrf2 mRNA* level was unchanged before and after MIND4-17 treatment in OB-6 cells (Figure 3C). Based on these results, we suggest that MIND4-17 treatment induces Nrf2′s departure from Keap1, leading to Nrf2 stabilization and accumulation. Thereafter, Nrf2 translocates to cell nuclei, and promotes transcription of multiple ARE-dependent anti-oxidant genes.

### MIND4-17 alleviates H_2_O_2_-induced oxidative stress in osteoblasts

**Figure 4:**
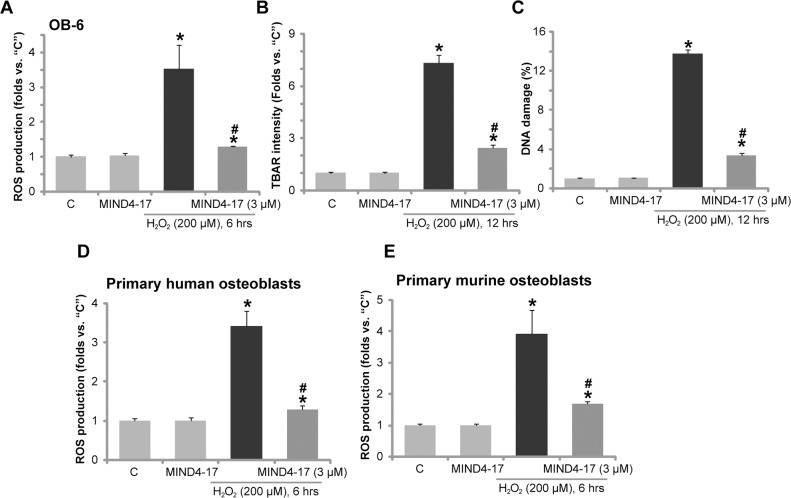
MIND4-17 alleviates H_2_O_2_-induced oxidative stress in osteoblasts OB-6 human osteoblastic cells (**A**-**C**), the primary human osteoblasts (**D**) or the primary murine osteoblasts (**E**) were pretreated for 1 hour with MIND4-17 (3 μM), following by hydrogen peroxide (“H_2_O_2_”, 200μM) treatment, cells were then cultured for the indicated time; Relative ROS production (DCFH-DA fluorescence intensity, A, D and E), lipid peroxidation (TBAR intensity, B)and DNA damages (p-γ-H2AX ratio, C). Data were presented as mean (n=5) ± standard deviation (SD). ^*^p<0.05 *vs.* “C” cells. ^#^*p*<0.05 *vs.* H_2_O_2_ only group. Experiments in this figure were repeated for three times, and similar results were obtained.

As shown in previous studies [18, 22, 30], H_2_O_2_ treatment in osteoblasts shall lead to reactive oxygen species (ROS) production, oxidative stresses, lipid peroxidation, DNA damages, and eventually cell death. In line with these findings, in OB-6 osteoblastic cells, H_2_O_2_ treatment (200 μM) in OB-6 osteoblastic cells induced profound ROS production, lipid peroxidation and DNA damages (Figure 4A-4C), which were tested by H2-DCFDA (2′,7′-dichlorofluorescein diacetate) fluorescence intensity OD (Figure 4A), TBAR (Thiobarbituric acid reactive substances) intensity (Figure 4B), phosphorylated-γ-H2AX percentage (Figure 4C), respectively. Remarkably, pretreatment with MIND4-17 (3 μM, 1 hour) largely attenuated such effects by the H_2_O_2_ (Figure 4A-4C). MIND4-17 treatment alone had no significant effect (Figure 4A-4C). In the primary human osteoblasts (Figure 4D) and primary murine osteoblasts (Figure 4E), pretreatment with MIND4-17 (3 μM, 1 hour) also largely inhibited H_2_O_2_-induced ROS production (DCFH-DA intensity increase). These results suggest that MIND4-17 significantly alleviated H_2_O_2_-induced oxidative stress in osteoblasts.

### Nrf2 knockdown abolishes MIND4-17-mediated cytoprotection against H_2_O_2_

**Figure 5:**
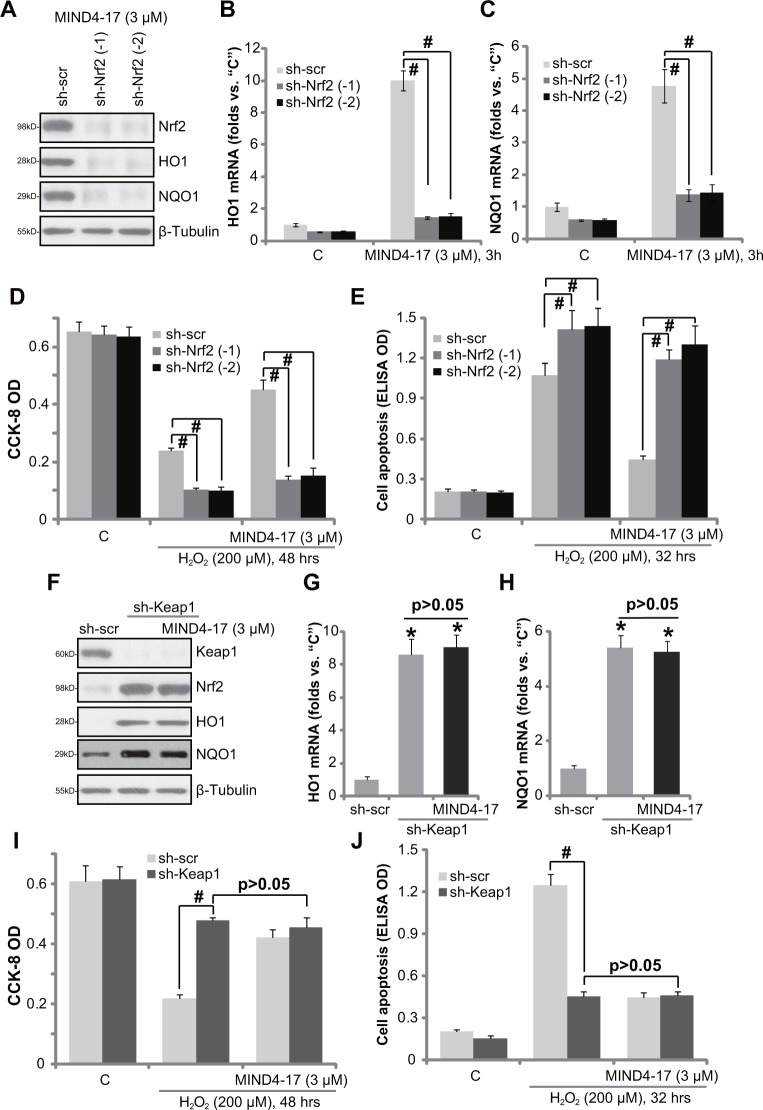
Nrf2 activation is required for MIND4-17-mediated cytoprotection OB-6 cells, stably expressing lentiviral scramble non-sense control shRNA (“sh-scr”) or Nrf2 shRNA (“sh-Nrf2, sequence -1/−2”) (**A**-**E**), as well as lentiviral Keap1 shRNA (“sh-Keap1”) (**F**-**J**), were treated with MIND4-17 (3 μM) for 3 hours, expressions of listed proteins were presented (A and F); mRNA expressions of listed genes were also shown (B, C, G and H); Cells were further treated with/out hydrogen peroxide (“H_2_O_2_”, 200 μM) for indicated time period, cell viability (CCK-8 assay, D and I) and apoptosis (Histone DNA ELISA assay, E and J) were also tested. For H_2_O_2_ experiments, MIND4-17 (3 μM) were pretreated before H_2_O_2_ for 1 hour. For all the assays, the exactsame number of viable cells with the applied shRNA was initially plated into each well. Data were presented as mean (n=5) ± standard deviation (SD). ^#^*p*<0.05. Experiments in this figure were repeated for three times, and similar results were obtained.

If Nrf2 activation is required for MIND4-17-mediated cytoprotection against H_2_O_2_, Nrf2 depletion should then abolish MIND4-17′s actions in osteoblasts. To test this hypothesis, we utilized short hairpin RNA (shRNA) method to inhibit Nrf2 expression in OB-6 cells. A set of two lentiviral shRNAs, against independent and non-overlapping human Nrf2 sequence (from Dr. Jiang [31]), were utilized. Puromycin was added to select the stable cells. Western blotting assay confirmed that the two applied Nrf2 shRNA (“-1/−2” [31]) each potently downregulated Nrf2 protein in MIND4-17 (3 μM, 3 hour)-treated OB-6 cells (Figure 5A), resulting in over 90% Nrf2 protein depletion (Figure 5A). Consequently, MIND4-17-induced protein (Figure 5A) and *mRNA* (Figure 5B and 5C) expressions of HO1 and NQO1 was also largely inhibited by Nrf2 shRNAs. CCK-8 assay results in Figure 5D and Histone DNA apoptosis ELISA assay results in Figure 5E displayed that H_2_O_2_-induced viability reduction and apoptosis were significantly boosted in Nrf2-silenced cells. More importantly, MIND4-17-induced anti-H_2_O_2_ activity was almost completely nullified in Nrf2 shRNA-expressing OB-6 cells (Figure 5D and 5E). These results indicate that Nrf2 activation is required for MIND4-17-mediated cytoprotection in OB-6 cells.

### Keap1 shRNA takes over MIND4-17′s actions in OB-6 cells

We speculated that Keap1 knockdown shall mimic MIND4-17′s actions in osteoblasts. Thus, as reported in our previous study [32], the lentiviral Keap1 shRNA (Santa Cruz Biotech) was employed to stably knockdown Keap1 in OB-6 cells. The applied Keap1 shRNA indeed significantly downregulated Keap1 protein in OB-6 cells (Figure 5F), which was followed by Nrf2 protein stabilization (Figure 5F), as well as protein (Figure 5F) and *mRNA* (Figure 5G and 5H) expressions of HO1 and NQO1. Similar to MIND4-17, Keap1 shRNA protected OB-6 cells from H_2_O_2_, leading to significantly less cell viability reduction (Figure 5I) and apoptosis induction (Figure 5J). Notably, adding MIND4-17 in Keap1-silnced OB-6 cells failed to further change Nrf2, Keap1 nor HO1 expressions (Figure 5F-5H). Neither did MIND4-17 offer extra protection against H_2_O_2_ (Figure 5I and 5J). Thus, Keap1 knockdown by targeted-shRNA mimicked and took over MIND4-17-mediated cytoprotection in OB-6 cells. These results again confirm that Nrf2 activation is required for MIND4-17-mediated cytoprotection in osteoblasts.

## DISCUSSION

Increased ROS production and subsequent oxidative stress is major cause of osteoblast cell injuries. Activation of Nrf2 signaling could efficiently protect osteoblasts [17, 33, 34] and other cells from oxidative stress. Keap1, a BTB-Kelch protein, is the upstream suppressor protein of Nrf2. Keap1 maintains Nrf2′s subcellular localization and steady-state level. It works as an E3 ubiquitin ligase complex with Nrf2, causing Nrf2 ubiquitin conjugation and proteasomal degradation [28, 35, 36].

Very recent studies have develop MIND4-17 as a highly-selective and unique small molecule activator of Nrf2 [19, 20]. By uniquely modifying Keap1′s sensor-cysteine C151 (also the oxidative stress reaction site) [19, 20], MIND4-17 induces Keap1 conformational change, causing Keap1-Nrf2 disassociation [19, 20]. Nrf2 will be away from the E3 ubiquitin ligase complex, causing its stabilization and activation, followed by de-novo transcription of Nrf2-dependent genes [19, 20]. In the present study, we showed that MIND4-17, at 1-10 μM, efficiently activated Nrf2 signaling in osteoblasts. MIND4-17 treatment induced Keap1-Nrf2 disassociation, causing Nrf2 stabilization, accumulation and nuclear translocation, and transcription of multiple key Nrf2 genes, including *HO1*, *NQO1*, *GCLC* and *GCLM* in osteoblasts/osteoblastic cells. Significantly, MIND4-17 potently inhibited H_2_O_2_-induced ROS production, lipid peroxidation and DNA damages, and significantly reduced following osteoblast cell apoptotic/non-apoptotic cell death.

In this study, we propose that activation of Nrf2 is required for MIND4-17-mediated cytoprotection against H_2_O_2_. Nrf2 knockdown by targeted shRNA exacerbated H_2_O_2_-induced cytotoxicity and death in OB-6 cells. Nrf2 shRNA almost nullified MIND4-17-mediated cytoprotective actions in OB-6 cells. Meanwhile, Keap1 shRNA mimicked MIND4-17′s actions and protected OB-6 cells from H_2_O_2_. Notably, MIND4-17 was in-effective in Keap1-silenced OB-6 cells. Therefore, Keap1 shRNA could take over MIND4-17′s actions in osteoblasts. These evidences clearly indicate that Nrf2 activation mediates MIND4-17-induced cytoprotection against H_2_O_2_ in osteoblasts.

## MATERIALS AND METHODS

### Chemicals, reagents and antibodies

MIND4-17 was synthesized by Minde Biotech (Suzhou, China), based on the structure in previous studies [19, 20]. H_2_O_2_ and puromycin were purchased from Sigma Chemicals (St. Louis, MO). Anti-Nrf2, Keap1, HO1, NQO1, GCLC, and GCLM, and β-Tubulin, Lamin-B antibodies were purchased from Santa Cruz Biotech (Santa Cruz, CA). Antibodies for cleaved-caspase-3 and cleaved-PARP were purchased from Cell Signaling Tech (Denver MA). All the mRNA primers were designed and provided by Genepharm (Shanghai, China).

### Culture of osteoblasts and osteoblastic cells

The OB-6 human osteoblastic cells were cultured and differentiated as described in our previous studies [37, 38]. The isolation and primary culture of murine osteoblasts derived from the trabecular bone of C57/B6 mice were described previously [39–42], with the animal protocol approved by Institutional Animal Care and Use Committee (IACUC) of Nanjing Medical University. C57/B6 mice, 5-6 week old, all female, were obtained from the Experimental Animal Center of Nanjing Medical University (Nanjing, China). The detailed protocol for the culture of primary human osteoblasts was also described previously. Briefly, the trabecular bone fragments were minced and were then digested (2 mg/mL collagenase type II, (300 U/mg; Sigma). The primary cells were thereafter placed in culture flasks with the described medium [43]. Medium was changed two-three times a week until cells reached confluence. The protocols using human tissues/cells were conducted according to the principles of Declaration of Helsinki, and were approved by the Ethics Review Board (ERB) of Nanjing Medical University.

### Cell survival and cell death assay

Cell Counting Kit-8 (CCK-8, Dojindo Laboratories, Kumamoto, Japan) was employed to test survival of osteoblasts/osteoblastic cells, as described previously [39, 40, 42]. Death of osteoblasts/osteoblastic cells was tested by measuring Lactate Dehydrogenase (LDH) release in the medium, using a two-step enzymatic reaction LDH assay kit (Takara, Tokyo, Japan) [39, 40, 42].

### Cell apoptosis assay

In line with our previous studies [39, 40], the histone-DNA ELISA plus kit (Roche, Palo Alto, CA) was utilized, and ELISA OD at 450 nm was recorded to quantitatively reflect cell apoptosis intensity [39, 40].

### Fluorescence activated cell sorting (FACS) assay

Osteoblasts/osteoblastic cells with applied treatment were washed and resuspended in binding buffer together with 2.5 μL of Annexin V-FITC (Invitrogen, Shanghai, China) and 2.5 μL of propidium iodide (PI) (Invitrogen). Osteoblasts/osteoblastic cells were then examined by flow cytometry using the CellQuest software (BD Biosciences, Shanghai, China). Annexin V positive cells were labeled as apoptotic cells [44, 45]. Annexin V negative and PI positive cells were labeled as non-apoptotic dead cells [44, 45].

### Western blotting assay

The detailed protocol of Western blotting assay has been extensively discussed in our previous studies [37, 39–42]. The protocol of isolation of nuclear proteins was described in detail in the previous studies [44, 46].

### Co-immunoprecipitation (Co-IP) assay

As described [44, 46], the Co-IP assay was performed to test the association between Keap1-Nrf2. In brief, OB-6 osteoblastic cells with applied treatment were lysed [44]. To the cleared lysates, 0.25 μg of Nrf2 antibody (Santa Cruz Biotech) was added per 0.8 mg of total cellular lysate proteins, and the immune complex formed by rotation for 24 hours at 4°C. The protein A/G-Sepharose (25 μL, Sigma) was then added and the incubation continued for additional 12 hours. The resulting immuno-precipitates captured with protein A/G-Sepharose were washed four times with CHAPS-containing buffer and analyzed by Western blotting assay.

### RNA isolation and qRT-PCR

Total RNA in osteoblasts/osteoblastic cells was extracted by the RNeasy Midi Kit (Qiagen, Wuxi, China). Five-hundred ng of total RNA per treatment was reverse-transcribed through the RT-PCR kit (TOYOBO, Japan). Quantitative real-time PCR (“qRT-PCR”) was performed through employing the SYBR green kit [47, 48], under the ABI-7600 Fast-PCR system (Applied Biosystems, Shanghai, China). The mRNA primers of Nrf2 pathways genes, including *Nrf2, HO-1*, *GCLC, GCLM* and *NQO1*, and GAPDH were previously described [31, 44, 46, 49]. GAPDH was always tested as the internal control gene. The 2^−ΔΔCt^ method was utilized to calculate relative expression of indicated *mRNA*. Its value was normalized to that of control cells.

### shRNA and stable cell selection

The two lentiviral Nrf2 shRNAs (“Seq-1/2”, with unique and non-overlapping sequences against human Nrf2) were provided by Dr. Jiang [31]. The shRNA-containing lentivirus was added to cultured osteoblasts/osteoblastic cells at 10 μL virus/1 mL medium. After 12 hours, puromycin (0.5 μg/mL) was added to select stable cells for additional 96 hours. Nrf2 knockdown in the resulting stable cells was verified by the qRT-PCR assay and Western blotting assay. Control cells were infected with lentiviral scramble control shRNA [37]. Keap1 shRNA and stable cell selection was described in the previous study [50].

### Detection of reactive oxygen species (ROS)

In line with our previous studies [18, 39, 41], ROS content in osteoblasts/osteoblastic cells was examined by the 2′,7′-dichlorofluorescein diacetate (H2-DCFDA; Abcam, Shanghai, China) intensity assay. ROS level (H2-DCFDA OD value) in treatment group was always normalized to that of control cells.

### Lipid peroxidation assay

Cellular TBAR (Thiobarbituric acid reactive substances) content was tested to examine the production of toxic aldehyde resulting from oxidative fatty acyl degradation, the malondialdehyde (MDA). TBAR intensity was then an indicator of lipid peroxidation. The detailed protocol for testing TBAR was described previously [51, 52]. The TBAR absorbance was measured at 532 nm. The lipid peroxide level was expressed as MDA/mg of protein. The value of treatment cells was normalized to that of control cells.

### γ-H2AX assay of DNA damages

Osteoblasts/osteoblastic cells with the indicated treatment were harvested, washed and incubated with a mouse monoclonal FITC-conjugated anti-phospho-γ-H2AX (at Ser139) antibody (Biyuntian, Wuxi, China). Cells were then subjected to FACS assay to determine the γ-H2AX percentage [53].

### Statistical analysis

Comparisons between groups were performed via one-way ANOVA and the Newman-Keuls test (SPSS 18.0). *p* values < 0.05 were considered statistically significant. Results of one set of experiments were shown in the Figures. Experiments in the Figures were repeated at least three times, and similar results were obtained.

## CONCLUSION

We conclude that MIND4-17 activates Nrf2 signaling and protects osteoblasts from H_2_O_2_. Dex and other glucocorticoids shall exert direct injuries to human osteoblasts, which is an important contributor of osteoporosis and osteonecrosis. The results of our study indicate that MIND4-17 and possible other Nrf2 signaling activators could possibly be further tested to treat Dex-associated bone damages.

## References

[1] Souttou B, Raulais D, Vigny M (2001). Pleiotrophin induces angiogenesis: involvement of the phosphoinositide-3 kinase but not the nitric oxide synthase pathways. J Cell Physiol.

[2] Himburg HA, Muramoto GG, Daher P, Meadows SK, Russell JL, Doan P, Chi JT, Salter AB, Lento WE, Reya T, Chao NJ, Chute JP (2010). Pleiotrophin regulates the expansion and regeneration of hematopoietic stem cells. Nat Med.

[3] Baek KH, Oh KW, Lee WY, Lee SS, Kim MK, Kwon HS, Rhee EJ, Han JH, Song KH, Cha BY, Lee KW, Kang MI (2010). Association of oxidative stress with postmenopausal osteoporosis and the effects of hydrogen peroxide on osteoclast formation in human bone marrow cell cultures. Calcif Tissue Int.

[4] Tare RS, Oreffo RO, Sato K, Rauvala H, Clarke NM, Roach HI (2002). Effects of targeted overexpression of pleiotrophin on postnatal bone development. Biochem Biophys Res Commun.

[5] Fan JB, Liu W, Zhu XH, Yuan K, Xu DW, Chen JJ, Cui ZM (2015). EGFR-AKT-mTOR activation mediates epiregulin-induced pleiotropic functions in cultured osteoblasts. Mol Cell Biochem.

[6] Herbst RS (2004). Review of epidermal growth factor receptor biology. Int J Radiat Oncol Biol Phys.

[7] Talasila KM, Soentgerath A, Euskirchen P, Rosland GV, Wang J, Huszthy PC, Prestegarden L, Skaftnesmo KO, Sakariassen PO, Eskilsson E, Stieber D, Keunen O, Brekka N (2013). EGFR wild-type amplification and activation promote invasion and development of glioblastoma independent of angiogenesis. Acta Neuropathologica.

[8] Liang D, Xiang L, Yang M, Zhang X, Guo B, Chen Y, Yang L, Cao J (2013). ZnT7 can protect MC3T3-E1 cells from oxidative stress-induced apoptosis via PI3K/Akt and MAPK/ERK signaling pathways. Cell Signal.

[9] Rigel DS, Friedman RJ, Kopf AW (1996). The incidence of malignant melanoma in the United States: issues as we approach the 21st century. J Am Acad Dermatol.

[10] Salopek TG, Marghoob AA, Slade JM, Rao B, Rigel DS, Kopf AW, Bart RS (1995). An estimate of the incidence of malignant melanoma in the United States. Based on a survey of members of the American Academy of Dermatology. Dermatol Surg.

[11] Koh HK (1991). Cutaneous melanoma. N Engl J Med.

[12] Suzuki T, Yamamoto M (2015). Molecular basis of the Keap1-Nrf2 system. Free Radic Biol Med.

[13] Nguyen T, Nioi P, Pickett CB (2009). The Nrf2-antioxidant response element signaling pathway and its activation by oxidative stress. J Biol Chem.

[14] Crunkhorn S (2012). Deal watch: Abbott boosts investment in NRF2 activators for reducing oxidative stress. Nat Rev Drug Discov.

[15] Tung MC, Lin PL, Wang YC, He TY, Lee MC, Yeh SD, Chen CY, Lee H (2015). Mutant p53 confers chemoresistance in non-small cell lung cancer by upregulating Nrf2. Oncotarget.

[16] Ning S, Sekar TV, Scicinski J, Oronsky B, Peehl DM, Knox SJ, Paulmurugan R (2015). Nrf2 activity as a potential biomarker for the pan-epigenetic anticancer agent, RRx-001. Oncotarget.

[17] Han D, Chen W, Gu X, Shan R, Zou J, Liu G, Shahid M, Gao J, Han B (2017). Cytoprotective effect of chlorogenic acid against hydrogen peroxide-induced oxidative stress in MC3T3-E1 cells through PI3K/Akt-mediated Nrf2/HO-1 signaling pathway. Oncotarget.

[18] Liu W, Mao L, Ji F, Chen F, Hao Y, Liu G (2017). Targeted activation of AMPK by GSK621 ameliorates H2O2-induced damages in osteoblasts. Oncotarget.

[19] Quinti L, Casale M, Moniot S, Pais TF, Van Kanegan MJ, Kaltenbach LS, Pallos J, Lim RG, Naidu SD, Runne H, Meisel L, Rauf NA, Leyfer D (2016). SIRT2- and NRF2-Targeting Thiazole-Containing Compound with Therapeutic Activity in Huntington's Disease Models. Cell Chem Biol.

[20] Quinti L, Dayalan Naidu S, Trager U, Chen X, Kegel-Gleason K, Lleres D, Connolly C, Chopra V, Low C, Moniot S, Sapp E, Tousley AR, Vodicka P (2017). KEAP1-modifying small molecule reveals muted NRF2 signaling responses in neural stem cells from Huntington's disease patients. Proc Natl Acad Sci U S A.

[21] Xu ZS, Wang XY, Xiao DM, Hu LF, Lu M, Wu ZY, Bian JS (2011). Hydrogen sulfide protects MC3T3-E1 osteoblastic cells against H2O2-induced oxidative damage-implications for the treatment of osteoporosis. Free Radic Biol Med.

[22] She C, Zhu LQ, Zhen YF, Wang XD, Dong QR (2014). Activation of AMPK protects against hydrogen peroxide-induced osteoblast apoptosis through autophagy induction and NADPH maintenance: New implications for osteonecrosis treatment?. Cell Signal.

[23] Fan JB, Ruan JW, Liu W, Zhu LQ, Zhu XH, Yi H, Cui SY, Zhao JN, Cui ZM (2016). miR-135b expression downregulates Ppm1e to activate AMPK signaling and protect osteoblastic cells from dexamethasone. Oncotarget.

[24] Halestrap AP (2006). Calcium, mitochondria and reperfusion injury: a pore way to die. Biochem Soc Trans.

[25] Halestrap A (2005). Biochemistry: a pore way to die. Nature.

[26] Baines CP, Kaiser RA, Purcell NH, Blair NS, Osinska H, Hambleton MA, Brunskill EW, Sayen MR, Gottlieb RA, Dorn GW, Robbins J, Molkentin JD (2005). Loss of cyclophilin D reveals a critical role for mitochondrial permeability transition in cell death. Nature.

[27] Wakabayashi N, Itoh K, Wakabayashi J, Motohashi H, Noda S, Takahashi S, Imakado S, Kotsuji T, Otsuka F, Roop DR, Harada T, Engel JD, Yamamoto M (2003). Keap1-null mutation leads to postnatal lethality due to constitutive Nrf2 activation. Nat Genet.

[28] Itoh K, Tong KI, Yamamoto M (2004). Molecular mechanism activating Nrf2-Keap1 pathway in regulation of adaptive response to electrophiles. Free Radic Biol Med.

[29] Furukawa M, Xiong Y (2005). BTB protein Keap1 targets antioxidant transcription factor Nrf2 for ubiquitination by the Cullin 3-Roc1 ligase. Mol Cell Biol.

[30] Zhu Y, Zhou J, Ao R, Yu B (2014). A-769662 protects osteoblasts from hydrogen dioxide-induced apoptosis through activating of AMP-activated protein kinase (AMPK). Int J Mol Sci.

[31] Gong YQ, Huang W, Li KR, Liu YY, Cao GF, Cao C, Jiang Q (2016). SC79 protects retinal pigment epithelium cells from UV radiation via activating Akt-Nrf2 signaling. Oncotarget.

[32] Cheng LB, Li KR, Yi N, Li XM, Wang F, Xue B, Pan YS, Yao J, Jiang Q, Wu ZF (2017). miRNA-141 attenuates UV-induced oxidative stress via activating Keap1-Nrf2 signaling in human retinal pigment epithelium cells and retinal ganglion cells. Oncotarget.

[33] Liu W, Mao L, Ji F, Chen F, Wang S, Xie Y (2017). Icariside II activates EGFR-Akt-Nrf2 signaling and protects osteoblasts from dexamethasone. Oncotarget.

[34] Li ST, Chen NN, Qiao YB, Zhu WL, Ruan JW, Zhou XZ (2016). SC79 rescues osteoblasts from dexamethasone though activating Akt-Nrf2 signaling. Biochem Biophys Res Commun.

[35] Kensler TW, Wakabayashi N, Biswal S (2007). Cell survival responses to environmental stresses via the Keap1-Nrf2-ARE pathway. Annu Rev Pharmacol Toxicol.

[36] Li W, Kong AN (2009). Molecular mechanisms of Nrf2-mediated antioxidant response. Mol Carcinog.

[37] Guo S, Chen C, Ji F, Mao L, Xie Y (2017). PP2A catalytic subunit silence by microRNA-429 activates AMPK and protects osteoblastic cells from dexamethasone. Biochem Biophys Res Commun.

[38] Fan JB, Liu W, Zhu XH, Yi H, Cui SY, Zhao JN, Cui ZM (2017). microRNA-25 targets PKCζ and protects osteoblastic cells from dexamethasone via activating AMPK signaling. Oncotarget.

[39] Ji F, Mao L, Liu Y, Cao X, Xie Y, Wang S, Fei H (2015). K6PC-5, a novel sphingosine kinase 1 (SphK1) activator, alleviates dexamethasone-induced damages to osteoblasts through activating SphK1-Akt signaling. Biochem Biophys Res Commun.

[40] Guo S, Xie Y, Fan JB, Ji F, Wang S, Fei H (2016). alpha-Melanocyte stimulating hormone attenuates dexamethasone-induced osteoblast damages through activating melanocortin receptor 4-SphK1 signaling. Biochem Biophys Res Commun.

[41] Guo S, Mao L, Ji F, Wang S, Xie Y, Fei H, Wang XD (2016). Activating AMP-activated protein kinase by an alpha1 selective activator compound 13 attenuates dexamethasone-induced osteoblast cell death. Biochem Biophys Res Commun.

[42] Zhao S, Chen C, Wang S, Ji F, Xie Y (2016). MHY1485 activates mTOR and protects osteoblasts from dexamethasone. Biochem Biophys Res Commun.

[43] van der Meijden K, van Essen HW, Bloemers FW, Schulten EA, Lips P, Bravenboer N (2016). Regulation of CYP27B1 mRNA Expression in Primary Human Osteoblasts. Calcif Tissue Int.

[44] Zhang H, Liu YY, Jiang Q, Li KR, Zhao YX, Cao C, Yao J (2014). Salvianolic acid A protects RPE cells against oxidative stress through activation of Nrf2/HO-1 signaling. Free Radic Biol Med.

[45] Yang L, Zheng LY, Tian Y, Zhang ZQ, Dong WL, Wang XF, Zhang XY, Cao C (2015). C6 ceramide dramatically enhances docetaxel-induced growth inhibition and apoptosis in cultured breast cancer cells: a mechanism study. Exp Cell Res.

[46] Li KR, Yang SQ, Gong YQ, Yang H, Li XM, Zhao YX, Yao J, Jiang Q, Cao C (2016). 3H-1,2-dithiole-3-thione protects retinal pigment epithelium cells against Ultra-violet radiation via activation of Akt-mTORC1-dependent Nrf2-HO-1 signaling. Sci Rep.

[47] Ji C, Huang JW, Xu QY, Zhang J, Lin MT, Tu Y, He L, Bi ZG, Cheng B (2016). Gremlin inhibits UV-induced skin cell damages via activating VEGFR2-Nrf2 signaling. Oncotarget.

[48] Jang HJ, Hong EM, Kim M, Kim JH, Jang J, Park SW, Byun HW, Koh DH, Choi MH, Kae SH, Lee J (2016). Simvastatin induces heme oxygenase-1 via NF-E2-related factor 2 (Nrf2) activation through ERK and PI3K/Akt pathway in colon cancer. Oncotarget.

[49] Li L, Zhu K, Liu Y, Wu X, Wu J, Zhao Y, Zhao J (2015). Targeting thioredoxin-1 with siRNA exacerbates oxidative stress injury after cerebral ischemia/reperfusion in rats. Neuroscience.

[50] Sun X, Ou Z, Chen R, Niu X, Chen D, Kang R, Tang D (2016). Activation of the p62-Keap1-NRF2 pathway protects against ferroptosis in hepatocellular carcinoma cells. Hepatology.

[51] Cortizo AM, Bruzzone L, Molinuevo S, Etcheverry SB (2000). A possible role of oxidative stress in the vanadium-induced cytotoxicity in the MC3T3E1 osteoblast and UMR106 osteosarcoma cell lines. Toxicology.

[52] Fernandez-Blanco C, Font G, Ruiz MJ (2014). Oxidative stress of alternariol in Caco-2 cells. Toxicol Lett.

[53] Ewald B, Sampath D, Plunkett W (2007). H2AX phosphorylation marks gemcitabine-induced stalled replication forks and their collapse upon S-phase checkpoint abrogation. Mol Cancer Ther.

